# Telestroke for the Treatment of Ischemic Stroke in Western China During the COVID-19 Pandemic: A Multicenter Observational Study

**DOI:** 10.3389/fneur.2021.822342

**Published:** 2022-01-31

**Authors:** Ning Chen, Xintong Wu, Muke Zhou, Rongdong Yang, Daofeng Chen, Ming Liao, Yongyi Deng, Zhen Hong, Dong Zhou, Li He

**Affiliations:** ^1^Department of Neurology, West China Hospital, Sichuan University, Chengdu, China; ^2^Department of Neurology, Guangyuan Central Hospital, Guangyuan, China; ^3^Department of Neurology, The Second People's Hospital of Yibin, Yibin, China; ^4^Department of Neurology, The First People's Hospital of Jintang, Chengdu, China; ^5^Department of Neurology, West China Ganzi Hospital of Sichuan University, Kangding, China

**Keywords:** coronavirus, Covid-19, telemedicine, telestroke, stroke, thrombolysis

## Abstract

**Background:**

Intravenous thrombolysis is still underutilized in patients with acute ischemic stroke (AIS) in China. A promising strategy for addressing this issue, especially in situations, such as the global pandemic of coronavirus disease 2019 (COVID-19), is the telestroke mode, which remains to be widely implemented in China. The present study aimed to assess the effects of telemedicine for patients with stroke in Western China, as well as the impact of the pandemic on telestroke services in 1 year after the COVID-19 outbreak.

**Methods:**

In this 2-year multicenter observational study, we retrospectively collected data from 10 hospitals within the Sichuan Telestroke and Telethrombolysis Network. Demographic and clinical characteristics of patients with IS and those relevant to thrombolysis were compared between the pre-telestroke and post-telestroke phases, and between the periods before and after declaration of the COVID-19 pandemic.

**Results:**

A total of 11,449 admissions with a primary diagnosis of IS were recorded during the study period. Prior to telestroke implementation, 6.7% of patients (*n* = 367) received intravenous thrombolysis, and the proportion increased to 7.4% (*n* = 443; *p* = 0.084) in the post-telestroke phase. The thrombolysis rate was 7.4% during the COVID-19 pandemic and in the latter half of the year when the viral spread was better controlled in China. The mean door-to-needle time (DNT) was significantly shorter after implementation of the telestroke network (63.76 ± 13.50 vs. 52.66 ± 25.49 min; *p* < 0.001).

**Conclusions:**

Telemedicine is effective in improving the thrombolysis administration among patients with IS in Western China. Implementation of the telestroke network should be promoted, especially when access to care is affected by public health emergencies, such as the COVID-19 pandemic.

## Introduction

Stroke is the third leading cause of global disease burden (1) and the leading cause of death in China (2). Ischemic stroke (IS) accounts for ~60–80% of all stroke cases (2, 3). Intravenous thrombolysis remains a standard hyperacute therapy for IS (4), but it is still underused in China. Less than 3% of patients received such treatment, a rate much lower than that in high-income countries (2). Although the patients arrived within 3.5 h after initial symptom onset, only 24.2% were treated with intravenous recombinant tissue plasminogen activator (rtPA) within 4.5 h (5). Due to the diversity of medical resources and regional economies, stroke control is still a major challenge in China (6). Arriving at rural, non-teaching hospitals without stroke centers is one of the most important factors limiting the use of acute treatments (7). Telemedicine is one way to address this issue. Indeed, telemedicine has been approved as an effective and safe for decision-making regarding thrombolysis and has been widely recommended worldwide (8).

Situations, such as the global pandemic of coronavirus disease 2019 (COVID-19), when social distancing has been implemented to contain the spread of the virus, further emphasize the benefits of telemedicine (9). However, a report from the Mayo Clinic indicated that there was a substantial decrease in telestroke activations after the WHO declared COVID-19 a pandemic. As the observation period of the study was short (30 days preceding to 30 days following the declaration), the causes of this reduction remain unclear (10).

Therefore, the present study aimed to assess the effects of telemedicine implementation for patients with stroke in Western China, and to analyze the impact of the pandemic on telestroke services in 1 year after the COVID-19 outbreak.

## Methods

### Study Design

In this 2-year multicenter observational study, we retrospectively reviewed and collected consecutive admissions of patients with a primary diagnosis of acute ischemic stroke (AIS) between January 2019 and December 2020 treated at 10 hospitals within the Sichuan telestroke and telethrombolysis network. AIS was defined in accordance with the WHO criteria (11, 12) or the Chinese guidelines for the clinical management of ischemic cerebrovascular diseases (13). The ethics committee of West China Hospital, Sichuan University approved the study [No.2021 (1723)], and waived the need for obtaining patient informed consent, since all data were retrospectively collected and individual information were not disclosed.

**Figure 1:**
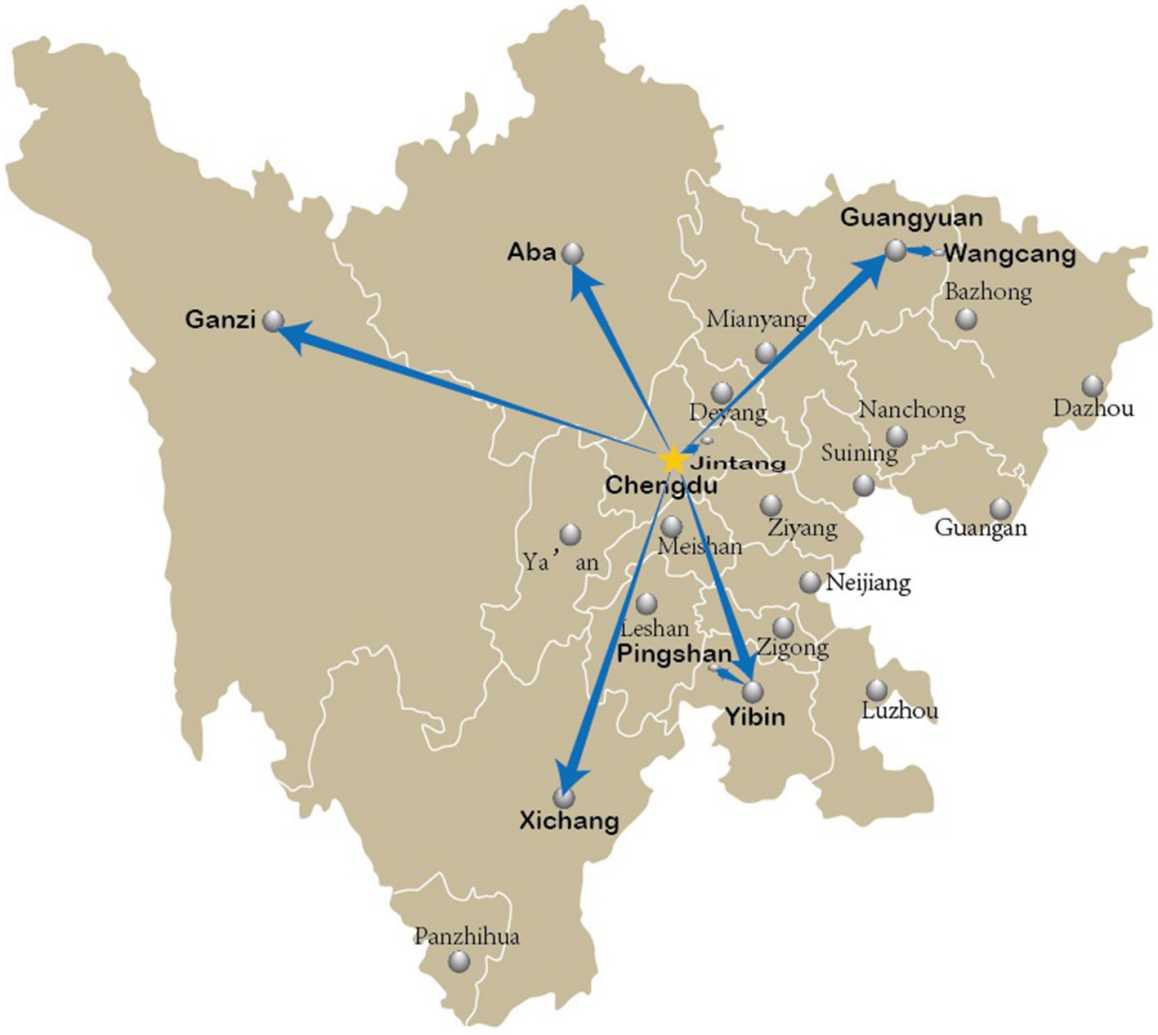
The pilot Telestroke and Telethrombolysis Network of Sichuan Province, China. West China Hospital of Sichuan University in Chengdu, the provincial capital, was designated as the hub center (golden star), and the network currently covers nine spoke hospitals (in boldface; two institutions in Pingshan, and one in each other region) around Sichuan.

The Sichuan telestroke and telethrombolysis network was established at the end of 2019, with support from the Sichuan Provincial Department of Science and Technology. This initiative aims to improve the use of intravenous thrombolysis in appropriate cases of AIS and to optimize the comprehensive stroke prevention and treatment in Sichuan province. It synergized roles of 4G/5G network, computer and smartphone applications, and the telestroke technology, based on the domestic and global evidence and experiences of telemedicine (9, 10, 14, 15). During the study period, this pilot network was composed of one academic hub center (West China Hospital of Sichuan University) and nine telemedically connected spoke hospitals located throughout the Sichuan Province in Western China (Figure 1). The mean distance between a spoke hospital and the hub is 270.6 km (range: 59–443 km). Using this network, telemedicine consultation for any candidate with possible AIS in each spoke hospital was instantly available 24 h per day and 7 days a week. Once the telestroke network was activated, the stroke experts in the hub center were able to view real-time images and data of each patient, and to supervise and guide local clinicians in spoke hospitals to do physical and neurological examinations and further assess the eligibility of patients for thrombolysis. Clinical information was recorded by each hospital and collected online. Online training sessions for the management of acute stroke were conducted regularly, to improve the professional level of local physicians in the management of stroke.

The study period was divided into two phases: the pre-telestroke phase (January–December 2019) and the post-telestroke phase (January–December 2020). Although the COVID-19 outbreak occurred in China at the beginning of 2020, the program continued to operate since most of the work could be conducted online after the establishment of the spoke network. To explore the impact of the COVID-19 pandemic on telestroke implementation, we further divided the post-telestroke study period into two phases: the first half of the year 2020 and the latter half, during which the epidemic was well-controlled in China. *Post-hoc* analysis was performed between the two phases.

### Statistical Analysis

Statistical analysis was performed using SPSS version 25 (IBM Corp., Armonk, NY, USA). Continuous variables are presented as the mean ± SD, while categorical variables are presented as frequencies and percentages. Differences between groups were assessed using the *t*-test for continuous variables and the chi-square test for categorical variables. Statistical tests were two-tailed, and a *p* < 0.05 was considered to be statistically significant in all analyses.

## Results

During the 2-year study period, a total of 11,449 admissions with a primary diagnosis of IS were recorded from all 10 hospitals. There was a slight increase in the total number of patients with IS from 2019 to 2020 (5,466 and 5,983 cases, respectively), and there was no significant difference in the mean number of cases occurring in each month. Approximately 60.5% of the patients were men, and the mean age and sex distribution were comparable between the study phases (Table 1).

**Table 1:** Characteristics of patients with ischemic stroke (IS) and those receiving intravenous thrombolysis.

	**Pre-telestroke phase (phase 1)**	**Post-telestroke phase (phase 2)**	**Post-telestroke phase, COVID-19 pandemic (phase 2-1)**	**Post-telestroke phase, COVID-19 better controlled in China (phase 2-2)**	***P*-value comparison between phase 1 and phase 2**	***P*-value comparison between phase 2-1 and phase 2-2**
Total number of patients with IS	5,466	5,983	2,811	3,172	\	\
Number of IS cases per month (mean ± SD)	456 ± 70.0	499 ± 78.8	469 ± 96.1	529 ± 47.6	0.869	0.199
Mean age (y, mean ± SD)	67.41 ± 4.87	67.49 ± 5.04	67.67 ± 4.71	67.32 ± 5.38	0.702	0.388
Male (*N*, %)	3,314 (60.6)	3,612 (60.4)	1,676 (59.6)	1,937 (61.1)	0.256	0.374
Thrombolysis treatment (*N*, %)	367 (6.7)	443 (7.4)	208 (7.4)	235 (7.4)	0.084	0.533
Mean DNT (min, mean ± SD)	63.76 ± 13.50	52.66 ± 25.49	51.98 ± 28.63	53.28 ± 22.40	<0.001	<0.001
Neurological improvement (NIHSS change^*^, mean ± SD)	2.13 ± 5.07	3.30 ± 4.11	3.47 ± 4.35	3.15 ± 3.90	0.005	0.491

* *NIHSS change: change of NIHSS score from admission to discharge. IS, ischemic stroke; SD, standard deviation; DNT, door-to-needle time; COVID-19, coronavirus disease 2019; NIHSS, the National Institutes of Health Stroke Scale*.

During the pre-telestroke phase, 6.7% of patients (*n* = 367) underwent intravenous thrombolysis, and the rate increased to 7.4% (*n* = 443) in the post-telestroke phase, although the difference between the two phases was not significant (*p* = 0.084). The thrombolysis rate was the same 7.4% during the COVID-19 pandemic and in the latter half of the year when the viral spread was better controlled in China. The mean door-to-needle time (DNT) was 63.76 ± 13.50 min among all patients who received thrombolytic treatment in the pre-telestroke phase. After the establishment and implementation of the telestroke network, the mean DNT was significantly decreased to 52.66 ± 25.49 min in all participating institutions (*p* < 0.001). The procedure seemed to be faster during the COVID-19 pandemic phase than during the better-controlled phase (51.98 ± 28.63 vs. 53.28 ± 22.40 min, *p* < 0.001) (Table 1). The short-term neurological improvement was evaluated by the change of National Institutes of Health Stroke Scale (NIHSS) score (16) from admission to discharge of each patient treated with intravenous rtPA. The NIHSS score decreased 2.13 ± 5.07 after treatment in the pre-telestroke phase, and a significantly better improvement was shown in the post-telestroke phase (NIHSS decreased 3.30 ± 4.11, *p* = 0.005) (Table 1).

**Figure 2:**
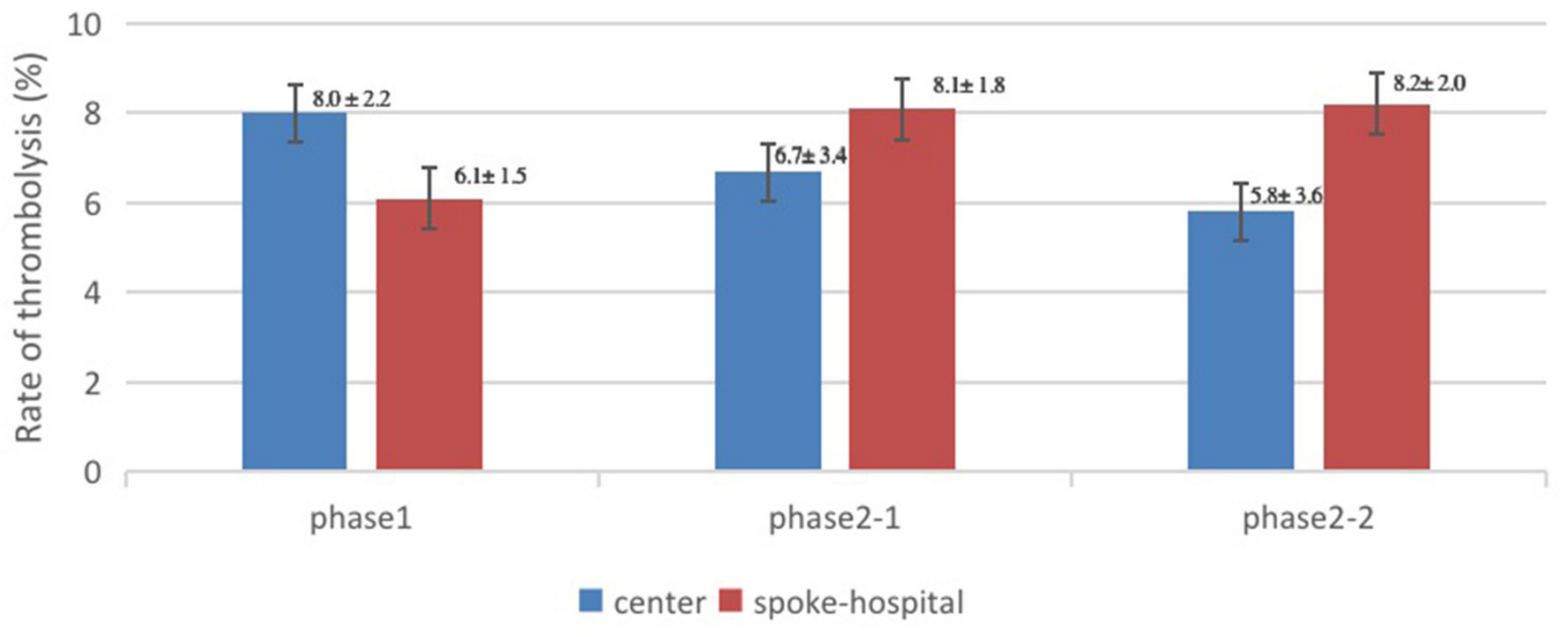
Comparisons of the thrombolysis rate between the hub center and spoke hospitals. Phase 1, pre-telestroke phase (January–December 2019); phase 2-1, post-telestroke phase during coronavirus disease 2019 (COVID-19) pandemic in China (January–June 2020); phase 2-2, post-telestroke phase, COVID-19 epidemic better controlled in China (July–December 2020).

We further compared thrombolysis rates between the hub center and the spoke hospitals. Figure 2 presents the comparisons and changes across the three phases. During the pre-telestroke phase, the monthly thrombolysis rate at West China Hospital was 8.0 ± 2.2%, while it was only 6.1 ± 1.5% in the other nine spoke hospitals (*p* = 0.174). In 2020, after the implementation of telestroke network in Sichuan, the thrombolysis rate in the spoke hospitals increased to 8.2 ± 1.8%, however, it decreased to 6.2 ± 3.3% in the hub center (*p* = 0.237). The proportions were similar for the two halves of 2020, and there were no significant differences between groups of hospitals (Figure 2).

## Discussion

In this study, we assessed the impact of telestroke implementation on the frequency and efficiency of thrombolysis treatment in patients with AIS in Sichuan Province, China. Our results indicated that the rate of intravenous thrombolysis increased after telestroke implementation, especially in the network spoke hospitals. Furthermore, the efficiency of treatment and neurological function improved significantly after the implementation of telestroke.

These findings are consistent with those of previous studies that have reported significant improvements in thrombolytic therapy after the implementation of telemedicine networks in other countries and territories (14, 17–19). Previous studies and systematic reviews have already verified the safety, efficacy, and reliability of intravenous rtPA delivery through telestroke networks (15); therefore, international guidelines for stroke recommend the use of telemedicine (8, 20). Our results support the promotion of such guidelines in Western China. Although the increase of intravenous thrombolysis rate was insignificant in this pilot study, there was an upward trend and an obvious improvement in the time efficiency, which is a key element for the early management of patients with AIS (8). In actual, the insignificance of thrombolysis increase might be mainly attributed to the data change in the hub center, which decreased after initiation of the telestroke program. This may be because West China Hospital is located in the city center of Chengdu, the capital of Sichuan province, and has the heaviest traffic conditions. Therefore, a relatively small number of patients with potential AIS can arrive within the 4.5-h time window. Instead, a number of patients only received thrombectomy in this tertiary medical center: some of them arrived beyond the 4.5-h window or had contraindications to thrombolysis, while others were transferred from other hospitals to receive the bridging therapy after intravenous rtPA. The rate of thrombectomy among all patients with AIS in West China Hospital was 9.3% (156/1,684) during the year 2020, an increase from the rate of 7.9% (149/1,891) in 2019. With new evidence regarding the benefit of endovascular thrombectomy alone in patients with AIS from large-vessel occlusion (21, 22), this rate may further increase in such central hospitals. Therefore, in the development and improvement of telestroke networks, attention should be directed toward consultations for the potential thrombectomy or bridging therapy and to the optimization of the transport process among hospitals.

We further assessed the impact of the COVID-19 pandemic on this newly established telemedicine network and found that operation of the system was hardly affected during this special circumstance. A slight growth in the volume of patient admissions was observed 1 year after the COVID-19 outbreak. This result differs from those reported in some prior studies, which noted obvious reductions in stroke admissions and a more than 25% drop in thrombolysis and thrombectomy procedures (23, 24). The Mayo Clinic reported a 50% reduction in total telemedicine activations for potential cases of stroke between 1 month before and 1 month after the declaration of the COVID-19 pandemic by the WHO (10). The main reason for this difference may be our relatively longer study period. We collected data for the full year after the COVID-19 outbreak. Importantly, in the latter half of the year, the epidemic has been better controlled across the country. The numbers of cumulative confirmed cases of COVID-19 in China were 83,534 and 3,537 in the first and second halves of the year, respectively. Among these, 595 and 258 cases occurred in Sichuan Province (data from the official website of the National Health Commission of China). We indeed observed a drop in stroke admissions in the first month of 2020 in our study, as several strict measures were implemented to contain the spread of the virus, which may have affected the rate of hospital visits. However, a stroke is a severe acute disease that must be given priority in the consultation and treatment. Following control of the epidemic in China, especially with the resumption of work and restoration of transportation, the total number of IS admissions rapidly increased and remained relatively stable. This increase is consistent with the trend of stroke incidence and prevalence in China in recent years (2, 5, 6). Furthermore, three spoke hospitals (located in Wangcang, Pingshan, and Ganzi) began to administer intravenous thrombolytic treatment for potential cases of AIS as the telestroke program progressed. These hospitals have thus received and cured more patients with stroke since the end of 2019.

Our findings showed higher rates of intravenous thrombolysis after implementation of the telestroke network, especially in the spoke hospitals. Sichuan Province, located in the southwest of China, covers an area of 485,000 km^2^ and has a population of more than 88 million. It is reputed as “a land of abundance,” but it is among the regions with the highest mortality-to-incidence ratio among patients with stroke in China (2, 3). Healthcare resources are relatively deficient in Western China, and highly qualified doctors and advanced technologies are often unavailable or severely lacking in rural and remote regions (9). Such imbalanced distribution of medical resources is present in several other regions of China (2). The long distance required for transfer to specialized centers may be the most important barriers to appropriate acute treatment, such as intravenous thrombolysis (7). Our present telestroke and telemedicine network includes regions throughout Sichuan in all directions, especially in areas with poor economic status (Figure 1). A convenient and effective medicine network can bring neurological expertise to the patient instead of requiring transfer of the patient to advanced hospitals, which can save time from symptom onset to the initiation of treatment. This telestroke treatment mode might be replicated and promoted in other regions with deficiency of medical resources. Online consultation and training can increase the knowledge and availability of a practitioner, allowing more patients to receive immediate, quality care close to home.

In the context of a pandemic or other public health emergency, telemedicine is advantageous given its ability to provide long-distance medical care and relieve local pressure (25). In our study, the rate of thrombolysis in patients with stroke was similar between the relatively severe phase and better-controlled phase of the COVID-19 epidemic, highlighting the stability of the telestroke network during the pandemic. Interestingly, the mean DNT was much shorter during the COVID-19 pandemic phase, possibly due to lower total patient volume in most hospitals and smoother access to investigations and treatments. However, the epidemic has not been resolved yet, meaning that the present data cannot perfectly capture the impact of COVID-19 on the implementation of the telestroke program in Sichuan. Our group will further collect and analyze relevant data following resolution of the pandemic.

Our study had some limitations. First, the data were retrospectively collected and reported by each hospital based on their clinical practice, so the quality of assessment and treatment may have varied among hospitals. Second, since some hospitals in the network had no special department of neurology, there were some missing or incomplete records in these sites before implementation of the telestroke program. Such data deficiency may have resulted in overestimation of the thrombolysis rate in the pre-telestroke phase (when they had not yet administered thrombolysis therapy), while underestimating the advantages of the telestroke network. The database established for the Sichuan telestroke program will allow for the collection of additional data that can be used to accurately reflect the effects of telestroke implementation in Western China. Third, we did not perform crosswise comparisons in the present study, meaning that the influence of other factors on improvements in AIS management cannot be ruled out. Furthermore, because of the limited data, we could not calculate the rate of thrombolysis among patients arriving within the time window or identify the reasons why they had not received thrombolytic treatment; therefore, the present study was unable to clarify the specific advantages of the telestroke network for patients with stroke.

Despite these limitations, the results of the current study support the implementation and promotion of telemedicine for patients with stroke in Western China, especially in special circumstance, such as the COVID-19 pandemic.

## Conclusion

Telemedicine is effective for improving the thrombolysis administration in patients with AIS in Western China. Implementation of the telestroke network should be promoted, especially when access to care is affected by public health emergencies, such as the COVID-19 pandemic.

## Data Availability Statement

The raw data supporting the conclusions of this article will be made available by the authors, without undue reservation.

## Author Contributions

NC, XW, MZ, RY, DC, ML, YD, ZH, DZ, and LH: acquisition, analysis, or interpretation of data for the work, drafting the work or revising it critically for important intellectual content, and final approval of the version to be published. All authors contributed to the article and approved the submitted version.

## Funding

The present study was supported by the Sichuan Science and Technology Program (2019YFH0196), the National Key Research and Development Program of China (2018YFC1311400), and the Project of Health Commission of Sichuan Province (19PJ048).

## Conflict of Interest

The authors declare that the research was conducted in the absence of any commercial or financial relationships that could be construed as a potential conflict of interest.

## Publisher's Note

All claims expressed in this article are solely those of the authors and do not necessarily represent those of their affiliated organizations, or those of the publisher, the editors and the reviewers. Any product that may be evaluated in this article, or claim that may be made by its manufacturer, is not guaranteed or endorsed by the publisher.
